# ELK1 Promotes Epithelial-Mesenchymal Transition and the Progression of Lung Adenocarcinoma by Upregulating B7-H3

**DOI:** 10.1155/2021/2805576

**Published:** 2021-12-21

**Authors:** Ting-ting Yu, Tao Zhang, Fei Su, Ying-long Li, Li Shan, Xiao-ming Hou, Ruo-zheng Wang

**Affiliations:** ^1^Department of Thoracic Oncology, Tumor Hospital Affiliated to Xinjiang Medical University, Urumqi 830011, China; ^2^Department of Oncology, The First Hospital of Lanzhou University, Lanzhou 730000, China; ^3^Radiation Therapy Center, Tumor Hospital Affiliated to Xinjiang Medical University, Urumqi 830011, China

## Abstract

In previous studies, we found that B7 homolog 3 (B7-H3) was highly expressed in lung adenocarcinoma (LUAD) and promoted epithelial-to-mesenchymal transition (EMT) of LUAD cells. However, the underlying molecular mechanism is unclear. This study is aimed at evaluating the role of Ets-like protein 1 (ELK1) as a transcriptional regulator of B7-H3 for mediating the development and progression of LUAD *in vitro* and *in vivo.* We confirmed that ELK1 is highly expressed in LUAD and is associated with poor patient prognosis. ELK1 was found to promote proliferation, invasion, migration, and EMT of LUAD cells through *in vivo* and *in vitro* experiments. In terms of mechanism, ELK1 binds to the B7-H3 promoter region and induces the upregulation of B7-H3 in LUAD. Our data suggest that ELK1 plays an important role in the development of LUAD and could be used as a prognostic marker and therapeutic target for LUAD.

## 1. Introduction

The lung cancer is becoming more and more common globally, which accounts for major causes resulting in cancer-associated mortality [1]. Lung adenocarcinoma (LUAD) is an important subtype of lung cancer, accounting for more than 40% of all lung cancers [2]. Although surgical techniques and treatments have been significantly improved, its 5-year survival rate remains poor [3]. The molecular mechanisms underlying the development and progression of LUAD are not fully understood. Therefore, the identification of LUAD-related markers is urgently needed.

Ets-like protein 1 (ELK1) is a member of the Ets family and the ternary complex factor subfamily. It is a transcriptional factor that plays a key role in modulating cell growth, differentiation, survival, and other biological behaviors [4]. Ets family proteins have been found to indirectly affect the expression of some tumor suppressors or oncogenes by regulating target gene promoters or through protein-protein interactions [5]. In cancer, ELK1 was confirmed to affect tumor cell proliferation and apoptosis [6]. For example, the expression of this protein is increased in bladder cancer and is responsible for enhancing tumor cell growth and invasion [7]. In hepatocellular carcinoma, ELK1 was confirmed to enhance malignancy by promoting the epithelial-to-mesenchymal transition (EMT) of tumor cells through increasing the aPKC-1 levels [8]. It has an important effect on breast and prostate cancer [9, 10]. In summary, ELK1 is tightly involved in tumor development. However, reports about its involvement in LUAD are not common, especially with respect to the associated mechanism.

In this study, we report that ELK1, as the transcriptional regulator of B7 homolog 3 (B7-H3; CD276), causes the latter to be upregulated in LUAD and further promotes malignant biological behaviors in cancer cells, in particular EMT. Our research provides a theoretical basis for ELK1 and B7-H3 to serve as potential targets for LUAD treatment.

## 2. Materials and Methods

### 2.1. Patient Samples

In the present study, we enrolled 90 LUAD patients undergoing resection at Tumor Hospital of Xinjiang Medical University from December 2010 to May 2013 with sufficient follow-up and clinical data. The clinical and pathological grade and tumor-node-metastasis (TNM) stage were based on the American Joint Committee on Cancer criteria. All obtained samples were subjected to formalin fixation and paraffin embedding. Fifty-eight males and thirty-two females were enrolled, and the average age was 60.3 ± 9.5 years (range, 46–79 years). Other clinicopathological features for all the 90 LUAD patients enrolled in this study can be found in Table 1. In addition, 4 fresh LUAD samples and 4 matched noncarcinoma samples were also harvested and stored at −80°C. These patients did not receive antitumor therapies. All enrolled patients signed informed consent. The Ethics Committee of Tumor Hospital Affiliated to Xinjiang Medical University approved this study (No. G-2020024).

### 2.2. Cell Lines and Culture

HEK293T cells, human bronchial epithelial (16HBE) cells, and LUAD cell lines, including NCI-H460, NCI-H292, PC-9, NCI-H1975, A549, NCI-H1299, and SPCA-1, were provided by cell bank of FuHeng Biology (Shanghai, China). The cell lines were cultured in Dulbecco's modified Eagle's medium (DMEM, Solarbio, Shanghai, China) supplemented with 10% fetal bovine serum (FBS) at 37°C conditions in an atmosphere containing 5% CO_2_.

### 2.3. Cell Transfection and Infection

Three short hairpin RNAs (shRNAs) targeting ELK1 and a negative control shRNA (sh-NC) were designed and synthesized by COMPASS BIOTECHNOLOGY (Quanzhou, Fujian, China). Table S1 displays shRNA sequences. The coding sequence of ELK1 or B7-H3 was cloned into the pLVX-puro plasmid (COMPASS BIOTECHNOLOGY), with the empty plasmid being the control (vector). The recombinant plasmids pMD2.G and psPAX2 were cotransfected into HEK293T cells using Lipofectamine 2000 Transfection Reagent (Invitrogen; Carlsbad, CA, USA) according to specific protocols. After 72 h, the virus was collected; specifically, the lentivirus supernatant liquid was collected, purified, and stored at −80°C. After LUAD cells were infected with lentivirus for 48 h, puromycin was used to screen for positive cells.

### 2.4. Quantitative Reverse Transcription Polymerase Chain Reaction (RT-qPCR)

TRIzol® (Invitrogen) was used for extracting total cellular RNA. Later, the cDNA synthesis kit (Invitrogen) was employed for preparing cDNA through reverse transcription in accordance with specific instructions. The SYBR® Green PCR Master Mix (Invitrogen) was used for qRT-PCR. Each experiment was repeated at least three times. *GAPDH* was chosen as the reference gene to quantify gene expression using the 2^−ΔΔCt^ method. Table S2 lists all the utilized primers.

### 2.5. Western Blotting

The radio immunoprecipitation assay (RIPA) lysis buffer (Beyotime, Shanghai, China) containing phosphatase and protease inhibitors (Solarbio; Beijing, China) was used for extracting total proteins from the cells. Later, the BCA Protein Assay Kit (Beyotime) was used for measuring the protein content. Then, sodium dodecyl sulfate-polyacrylamide gel electrophoresis was conducted to separate proteins, followed by transfer onto polyvinylidene fluoride (PVDF) membranes (Bio-Rad Laboratories, Inc., NY, USA). Subsequently, the membranes were blocked for 1 h using 5% defatted milk prepared in Tris-buffered saline. Then, the membranes were first incubated overnight with the primary antibodies at 4°C and then incubated for 2 h with the secondary antibodies at ambient temperature. Finally, images were acquired using the enhanced chemiluminescence system (Bio-Rad, Hercules, CA, USA). The following primary antibodies were used: anti-Slug (1: 1500), anti-Snail (1: 1000), and anti-B7-H3 (1: 1200) (all from Cell Signaling Technology; CST, Danvers, MA, USA); anti-ELK1 (1: 1000), anti-N-cadherin (1: 800), anti-Twist (1: 1000), and anti-GAPDH (1: 2000) (all from Abcam, Cambridge, MA, USA); and anti-E-cadherin (1: 1000) and anti-*β*-catenin (1: 1000) (both from Santa Cruz Biotechnology, Dallas, TX, USA).

### 2.6. Immunohistochemistry

A tissue microarray (TMA) was generated using tissue cores 2 mm in diameter, which were collected from the 90 LUAD patients described previously herein. First, 3% hydrogen peroxide was used to block the TMA for a period of 10 min, followed by incubation with the anti-ELK1 antibody (1 : 500, Abcam). In accordance with the method described in a previous study [11], ELK1 staining was assessed based on the staining intensity (1+, 2+, 3+, 4+, and 5+, which indicated that <10%, 10–25%, 26–50%, 51–75%, and >75% of cells were positive for nuclear staining, respectively). Scores of 1+ and 2+ indicated ELK1-low, whereas 3+, 4+, and 5+ indicated ELK1-high.

Immunostaining was performed using the diaminobenzidine substrate kit (USEN Biotechnology; Shanghai, China) in accordance with specific instructions provided along with the kit. Antibodies against PNCA (1: 400; Abcam) and Ki-67 (1: 200; Abcam) were used. 3,3′-Diaminobenzidine (DAB) was used to stain the sections for a period of 5 min, followed by hematoxylin incubation for 3 min and subsequently analyzed using an inverted microscope (Olympus IX53; Tokyo, Japan). Additional primary antibodies utilized in the present study included anti-N-cadherin (1: 200; Abcam), anti-E-cadherin (1: 500; Santa Cruz), anti-*β*-catenin (1: 200; Santa Cruz), anti-Snail (1: 100; CST), anti-Slug (1 : 200; CST), and anti-Twist (1: 400; Abcam).

### 2.7. Cell Counting Kit-8 (CCK-8) Assay

After 24 h of incubation, LUAD cells (5 × 10^3^ cells) were washed with PBS and exposed to CCK-8 solution (10 *μ*L, Dojindo, Tokyo, Japan). The microplate reader (BioTek, Winooski, VT, USA) was used for measuring absorbance (OD) at 450 nm.

### 2.8. 5-Ethynyl-2′-deoxyuridine (EdU) Assay

The EdU assay was performed using an EdU kit (Ribobio, Guangzhou, China). EdU (50 *μ*M) was used to treat LUAD cells for a period of 12 h. Next, 100 *μ*L Hoechst 33342 (Ribobio) and 1× Apollo solution were added to the cells. EdU activity in the cells was investigated using a fluorescence microscope (Olympus; Tokyo, Japan).

### 2.9. Wound Healing Assay

LUAD cells (1 × 10^5^) were seeded into 6-well plates and cultured for 24 h. Later, a scratch was made at the center of each well using a micropipette tip. Images of the wound were acquired at 0 and 24 h using an inverted microscope (Olympus).

### 2.10. Transwell Assay

Transwell chambers (Corning, New York, USA) were used to perform the cell invasion assay. Approximately 1 × 10^5^ LUAD cells were seeded in the top chamber and incubated for 24 h. The Transwell chamber was coated with Matrigel (Corning) to simulate the matrix. Complete medium was added to the bottom chamber of the Transwell chamber. Afterward, 4% polymethanol was used to fix the cells and 0.1% crystal violet was used for staining cells. Finally, the cells were observed under a microscope (Olympus).

### 2.11. Xenograft Tumors in Nude Mice

In this study, the animal experiments strictly followed the Animal Experiment Guidelines. The Ethics Committee of Tumor Hospital Affiliated to Xinjiang Medical University approved our study protocols (No. IACUC-20200331-22). The role of ELK1 in LUAD cell oncogenicity was examined *in vivo* after subcutaneous injection with transplanted cells (1 × 10^6^ per mouse) in the right armpit of mice. For this, 4–6-week-old BALB/c nude mice were randomly placed in one of the 4 groups, i.e., stable ELK1-silenced (sh-ELK1), lenti-virus negative control (sh-NC), stable ELK1-overexpression (ELK1), and empty plasmid (vector). The growth of the transplanted tumor was assessed based on bioluminescence images obtained using the IVIS Spectrum imaging system (PerkinElmer, Massachusetts, USA). For quantifying the bioluminescence signals *in vivo*, D-luciferin (150 mg/kg; Promega, Madison, Wisconsin, USA) was injected intraperitoneally. Every two days, we measured the tumor length and width to determine the tumor volume using the following equation: volume = width^2^ × length × 0.5.

### 2.12. Chromatin Immunoprecipitation (ChIP)

For the ChIP assay, the ChIP kit from Millipore (Billerica, MA, USA) was used. Briefly, after fixing the cells with 1% formaldehyde, the reaction was stopped using glycine. The cell lysate was placed in an ultrasonic disruptor to generate DNA fragments in the size range of 100–1000 bp. PCR was used to analyze the resulting precipitated DNA samples using the following primers: 5′-GGTTAACTCGACTGCGAGGA-3′ (sense) and 5′-CCAAGGCCACTAGGTGTCAG-3′ (antisense). Finally, PCR products were analyzed using agarose gel electrophoresis.

### 2.13. Luciferase Reporter Assays

In accordance with specific protocols, mutant (MUT) and wild-type (WT) B7-H3 promoter fragments were amplified and cloned into a pGL3 vector (Promega) to perform luciferase experiments. 293 T cells were seeded into a 24-well plate and then were cotransfected with pGL3-WT B7-H3, pGL3-MUT B7-H3, or pGL3 plasmids. After 48 h, the dual-luciferase reporter assay system (Promega) was used to assess luciferase activities (normalized to *Renilla*).

### 2.14. Bioinformatic Analysis

We downloaded RNA-seq data corresponding to LUAD samples from the Cancer Genome Atlas (TCGA; https://portal.gdc.cancer.gov/) database, which contains 535 LUAD samples and 59 normal samples. Transcription factors with the ability to bind to the B7-H3 promoter were searched using ALGGEN PROMO (http://alggen.lsi.upc.es/home.html) [12]. The JASPAR database (https://jaspar.genereg.net/) was used to predict the binding sites between ELK1 and B7-H3 promoters [13]. Kaplan-Meier Plotter (https://kmplot.com/analysis/) was used to assess the correlation between ELK1 expression and LUAD prognosis [14].

Possible biological pathways related to ELK1 expression were evaluated using gene set enrichment analysis (GSEA) [15]. The criteria included a normalized false discovery rate and enrichment score, which were used to identify significant differences in GSEA. Gene terms conforming to ∣logFC | ≥0.2 and *p* value < 0.05 were considered significant.

### 2.15. Statistical Analysis

Results were expressed as the mean ± SD. Statistical analysis was conducted using the SPSS software (Version 21.0; IBM, Chicago, USA). Student's *t*-test was used to compare the differences between two groups. For comparing multiple groups, one-way ANOVA was used. Outcome analysis for patients was conducted using Kaplan-Meier analysis, with overall survival (OS) being the primary outcome measure. *p* < 0.05 indicated significance. All experiments were performed three times.

## 3. Results

### 3.1. ELK1 Upregulates B7-H3 Level through the Combination with Its Promoter

**Figure 1:**
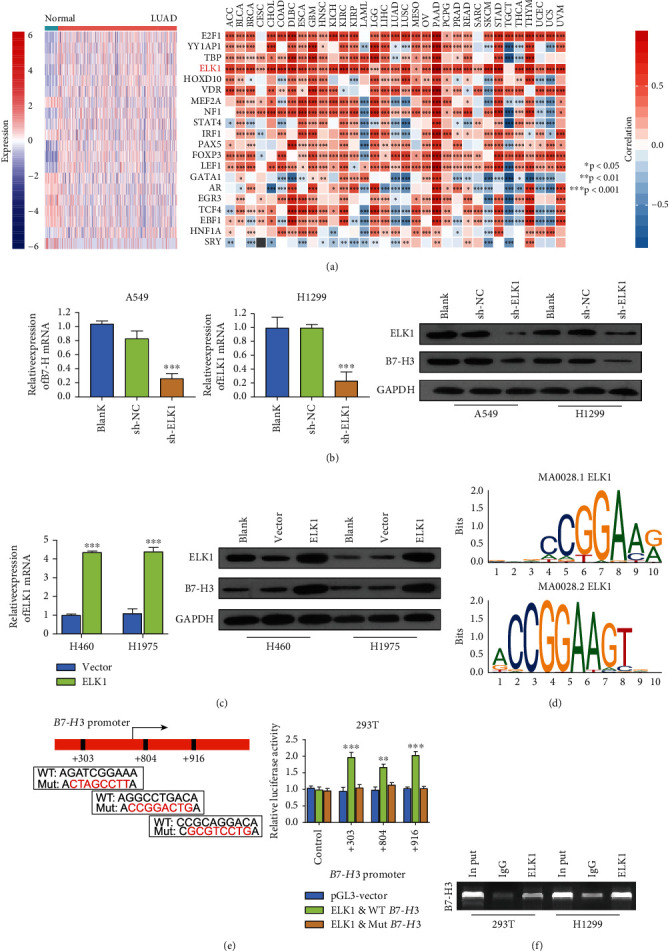
ELK1 is an important transcriptional regulator of B7-H3. (a) Heat map of expression of transcription factors of B7-H3 in LUAD samples and normal samples (left); analysis of the correlation between the expressions of ELK1 and B7-H3 in pan-cancer (right). (b) Knockdown of ELK1 inhibited the expression levels of B7-H3 mRNA and protein in A549 or H1299 cell lines. (c) Overexpression of ELK1 promoted the expression of B7-H3 mRNA and protein in H460 or H1975 cell lines. (d) Motif of promoter sequence of B7-H3 can bind to ELK1. (e) Schematic diagram of the B7-H3 promoter, including the predicted ELK1-binding regions (left) (the wild-type (WT) or mutant (MUT)) and binding site of B7-H3 which was synthesized for the luciferase reporter assay in 293 T cell lines (right). (f) ChIP analysis was used to determine the binding of ELK1 to the B7-H3 promoter, IgG as a negative control. ^∗^*p* < 0.05, ^∗∗^*p* < 0.01, and ^∗∗∗^*p* < 0.001.

In our previous study, B7-H3 upregulation in LUAD was in direct proportion to cancer development and indicated dismal prognosis [16]. However, the cause of the B7-H3 upregulation in LUAD remained unclear. To elucidate the potential mechanism underlying the effect of B7-H3 on LUAD, we predicted its interacting partners and cognate transcriptional pathways by analyzing the correlation with transcription factor expression in LUAD. First, we searched for transcriptional regulatory factors that can promote the expression of B7-H3 using the PROMO. Next, we analyzed the levels of these transcription factors and their correlations with B7-H3 expression in LUAD using RNA-seq data from TCGA. According to Figure 1(a), ELK1 was upregulated in LUAD samples and significantly and positively correlated with the expression of B7-H3. Therefore, we deduced that the role of B7-H3 in LUAD could be demonstrated by examining a factor that might regulate its transcription, i.e., ELK1.

Toward this, we tested the regulatory relationship between ELK1 and B7-H3. After knocking down or overexpressing ELK1, we analyzed the alterations in the expression of B7-H3 at the protein and mRNA levels within LUAD cells. Results consistent with those obtained using western blotting and RT-qPCR assays were observed, i.e., inhibition of ELK1 remarkably downregulated the expression of B7-H3 in the LUAD cell lines, and overexpression of ELK1 promoted the expression of B7-H3 (Figures 1(b) and 1(c)). To further identify the potential transcriptional regulatory relationship between ELK1 and B7-H3, we identified the predicted site on the B7-H3 promoter that could be bound by ELK1 using JASPAR (Figure 1(d)). Based on this finding, we considered that ELK1 is an effective transcription factor for B7-H3, and we decided to further verify the use of 293T cells. Once again, we used PROMO for analyzing three candidate bindings sites of ELK1 protein within B7-H3 promoter region (Figure 1(e)), and these three sites were mutated. We mutated the predicted ELK1-binding sites (+303, +804, and +916) on the B7-H3 sequence, and luciferase activity assays showed that the transcriptional activity of B7-H3 increased significantly after transfection with the ELK1-overexpression plasmid, but luciferase activity was enhanced by transfecting the mutant B7-H3 sequence (Figure 1(e)). Next, we investigated whether ELK1 could bind to the B7-H3 promoter and performed ChIP analysis in 293 T cells and H1299 cells. Compared to the negative control, i.e., normal rabbit IgG, the relative enrichment of the B7-H3 promoter was significantly increased upon immunoprecipitating with the ELK1 antibody (Figure 1(f)). ELK1 could bind to the promoter region of the B7-H3 locus in the aforementioned cells. In short, ELK1 promotes the transcription of B7-H3 by binding to its promoter.

### 3.2. ELK11 Is Upregulated in LUAD and Predicts Dismal Prognostic Outcomes of the Patients

**Figure 2:**
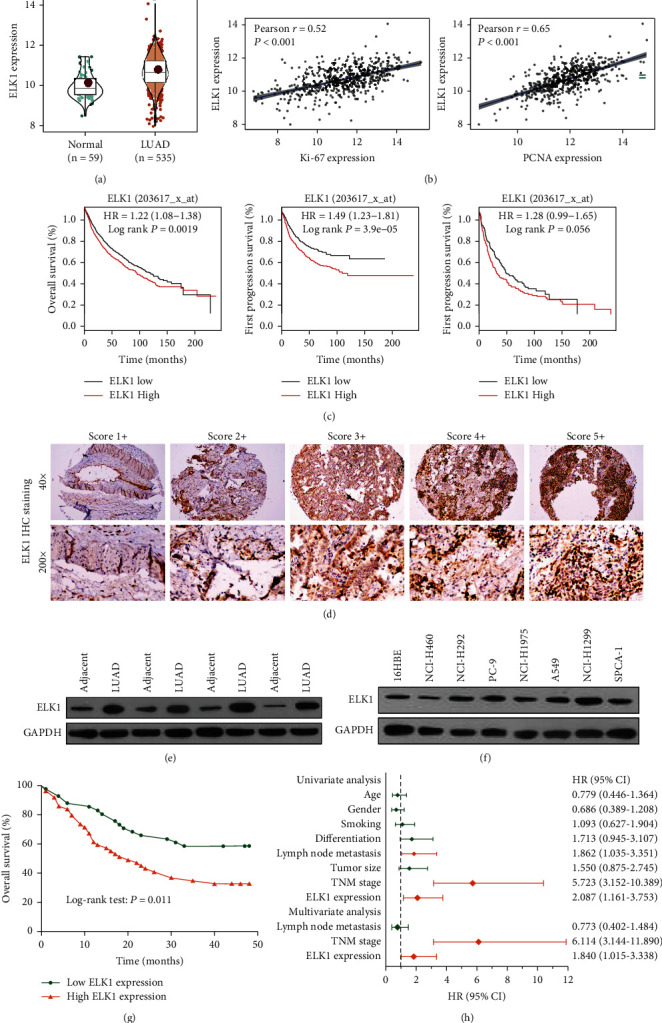
ELK1 is highly expressed in LUAD and indicates a poor prognosis for patients. (a) Analyze the expression of ELK1 in LUAD samples and normal samples based on TCGA-LUAD data. (b) Based on TCGA-LUAD data, the expression correlation between ELK1 and Ki-67 or PNCA was analyzed. (c) Kaplan-Meier survival analysis of LUAD patients indicates that the increase in ELK1 expression is correlated with poor prognosis. (d) Detection of ELK1 protein expression in LUAD tissues by IHC. (e) Quantitative analysis of the expression level of ELK1 protein by western blotting in four pairs of fresh LUAD and corresponding adjacent tissues. (f) Analyze the expression level of ELK1 protein in LUAD cell lines by western blotting. (g) Survival analysis of 90 LUAD patients shows that the high ELK1 expression is related to poor prognosis. (h) Univariate and multivariate analysis of overall survival of 90 LUAD patients. ^∗^*p* < 0.01.

To study the function of ELK1 in the development of LUAD, we analyzed it based on TCGA-LUAD-RNA-seq data. We predicted that ELK1 levels within the LUAD samples markedly increased relative to those in the normal samples (Figure 2(a)), and this was in direct proportion to the levels of Ki-67 or PCNA (Figure 2(b)). As revealed by Kaplan-Meier plotter survival analysis, high ELK1 expression indicates worse prognosis for patients with LUAD (Figure 2(c)). These results suggested that ELK1 might have an important function in enhancing LUAD progression.

To verify these results, ELK1 protein levels within LUAD cells and tissues were measured using IHC and western blot analysis. IHC showed that ELK1 was mostly expressed in the nuclei of LUAD cells. We scored the level of ELK1 expression based on the intensity of staining (range, 1–5; Figure 2(d)). Next, we quantitatively analyzed the expression of ELK1 protein in four pairs of LUAD and adjacent tissues. According to Figure 2(e), ELK1 levels within the LUAD samples usually increased relative to those in the matched noncarcinoma samples. Compared to those in normal tracheal epithelial cells, ELK1 levels within LUAD cells were markedly increased, especially in H1299 and A549 cells (Figure 2(f)).

For analyzing the association between ELK1 and prognosis of LUAD, 90 LUAD patients in the TMA cohort were classified as high or low ELK1 based on the IHC score. The results showed that ELK1 upregulation was tightly associated with the TNM stage, distant metastasis, and lymph node metastasis in patients with LUAD (*p* < 0.05), but not with sex, age, tumor size, smoking history, or differentiation degree (*p* > 0.05; Table 1). Kaplan-Meier analysis revealed that LUAD patients showing high ELK1 levels had markedly dismal OS relative to those showing low expression (Figure 2(g)). As shown in Figure 2(h), lymph node metastasis, TNM stage, and high ELK1 level were identified as the factors that independently predict LUAD prognosis.

### 3.3. ELK1 Promotes the Malignant Behavior of LUAD Cells *In Vitro*

**Figure 3:**
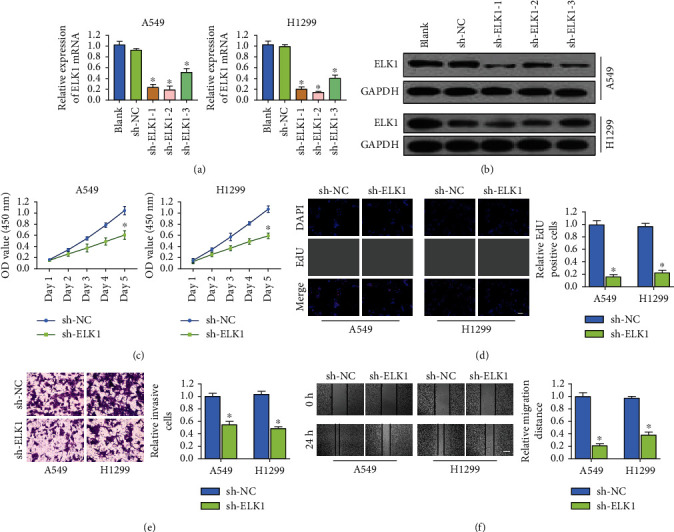
Knockdown of ELK1 inhibited malignant biological behavior of LUAD cell. (a, b) RT-qPCR and western blotting verified the knockdown efficiency of ELK1 in A549 or H1299 cell lines, respectively. (c, d) CCK-8 and EdU assays showed that ELK1 knockdown reduced the proliferation activity of A549 or H1299 cells, respectively (scale bar, 200 *μ*m). (e) Transwell analysis suggested that knockdown of ELK1 inhibited the invasion ability of A549 or H1299 cells (scale bar, 50 *μ*m). (f) Wound healing assay showed that knockdown of ELK1 inhibited the migration ability of A549 or H1299 cells (scale bar, 500 *μ*m). ^∗^*p* < 0.001.

For investigating the role of ELK1 in determining the biological behavior of LUAD cells, we designed three lentivirus-mediated shRNAs to knockdown ELK1 in H1299 and A549 cells. ELK1 knockdown was verified through western blotting and qRT-PCR (Figures 3(a) and 3(b)). Next, we performed EdU and CCK-8 assays for evaluating the effect of ELK1 on the proliferation of LUAD cells. Our results revealed that ELK1 knockdown remarkably suppressed the growth and activity of H1299 and A549 cells (Figures 3(c) and 3(d)). At the same time, Transwell assay was conducted. The results revealed that the knockdown of ELK1 significantly suppressed the invasiveness of LUAD cells (Figure 3(e)). Scratch assay was conducted for analyzing the effect of ELK1 on the migration ability of LUAD cells. According to our results, ELK1 knockdown suppressed cell migration (Figure 3(f)).

**Figure 4:**
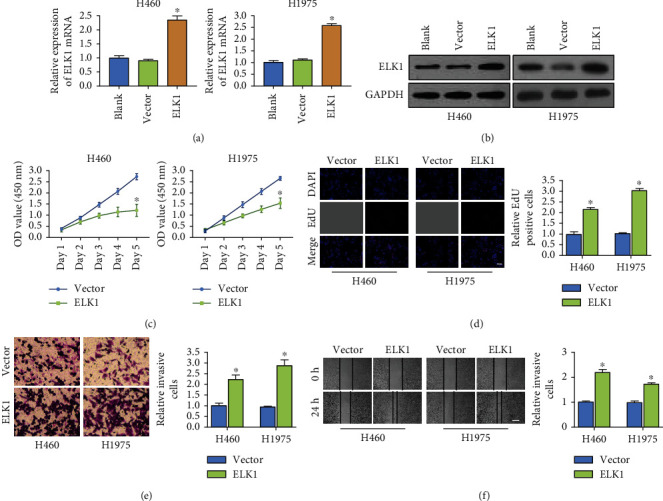
Overexpression of ELK1 promoted malignant biological behavior of LUAD cell. (a, b) RT-qPCR and western blotting verified the overexpression efficiency of ELK1 in H460 or H1975 cell lines, respectively. (c, d) CCK-8 and EdU assays showed that ELK1 overexpression promoted the proliferation activity of H460 or H1975 cells, respectively (scale bar, 200 *μ*m). (e) Transwell analysis suggested that overexpression of ELK1 promoted the invasion ability of H460 or H1975 cells (scale bar, 50 *μ*m). (f) Wound healing assay showed that overexpression of ELK1 promoted the migration ability of H460 or H1975 cells (scale bar, 500 *μ*m). ^∗^*p* < 0.001.

As shown in our previous results, ELK1 was expressed at the lowest level in H460 and H1975 cell lines. Therefore, we overexpressed ELK1 in the aforementioned LUAD cell lines (Figures 4(a) and 4(b)). We once again analyzed the effect of ELK1 on the growth, migration, and invasion of LUAD cells. Now, ELK1 overexpression enhanced the viability of LUAD cells and promoted their invasion and migration (Figures 4(c)–4(f)). Taken together, ELK1 promotes the malignant behavior of LUAD cells.

### 3.4. ELK1 Promotes LUAD Cell Proliferation *In Vivo*

**Figure 5:**
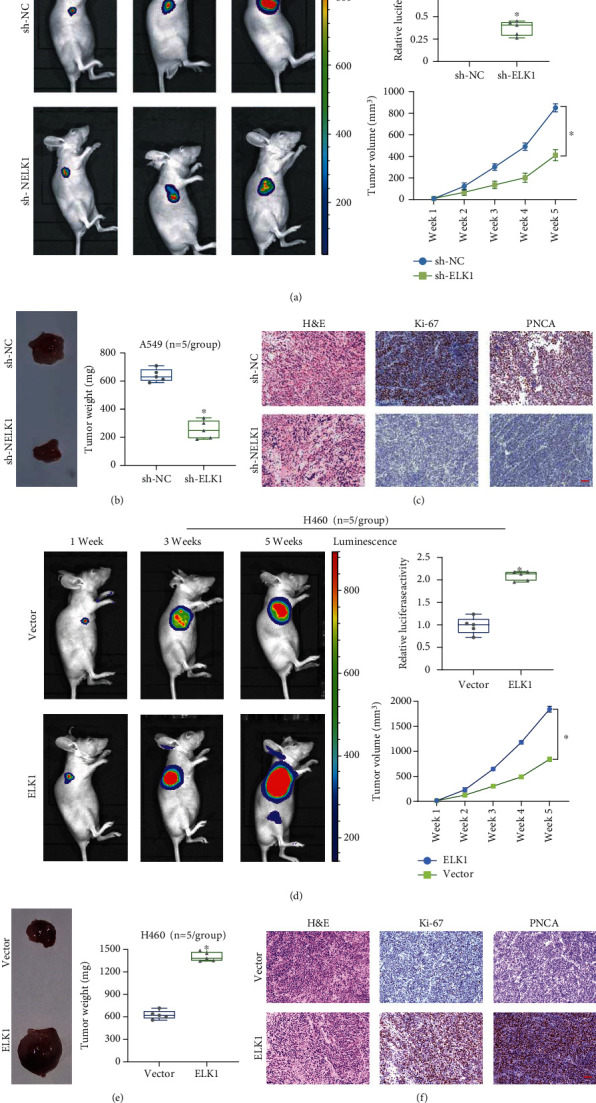
ELK1 promoted tumor growth of LUAD *in vivo*. (a) A549 cells (sh-NC vs. sh-ELK1) were injected subcutaneously in nude mice and representative bioluminescence image of LUAD mouse models for the detection of tumor growth at different time nodes. Knockdown of ELK1 significantly inhibited tumor growth in nude mice. (b) After five weeks, the tumors in the nude mice were removed and weighed. Knockdown of ELK1 significantly inhibited tumor weight in nude mice. (c) H&E staining and IHC evaluated the expression changes of Ki67 or PNCA after ELK1 knockdown (scale bar, 50 *μ*m). (d) H460 cells (vector vs. ELK1) were injected subcutaneously in nude mice and representative bioluminescence image of LUAD mouse models for the detection of tumor growth at different time nodes. Overexpression of ELK1 promoted tumor growth in nude mice. (e) After five weeks, the tumors in the nude mice were removed and weighed. Overexpression of ELK1 promoted tumor weight in nude mice. (f) H&E staining and IHC evaluated the expression changes of Ki67 or PNCA after ELK1 overexpression (scale bar, 50 *μ*m). ^∗^*p* < 0.001.

Nude mice were injected with ELK1-knockdown or ELK1-overexpressing LUAD cells via the armpits, for evaluating the effect of ELK1 on *in vivo* tumor growth. We used A549 cells to generate ELK1-knockdown mouse xenograft models (sh-ELK1 and sh-NC) and H460 cells to generate ELK1-overexpression mouse xenograft models (ELK1 and vector). The knockdown of ELK1 inhibited tumor growth in nude mice and remarkably reduced the tumor weights and volumes (Figures 5(a) and 5(b)). In contrast, in the mice administered with ELK1-overexpressing H460 cells, tumors developed faster compared to those in mice injected with the control, i.e., H460 cells (vector group), and exhibited higher tumor weights and larger tumor volumes (Figures 5(d) and 5(e)). Hematoxylin-eosin staining revealed structural destruction of the tumor tissue in xenotransplanted nude mice (Figures 5(c) and 5(f)). IHC assay revealed that ELK1 knockdown suppressed the expression of Ki-67 and PNCA (Figure 5(c)), whereas its overexpression had the opposite effect (Figure 5(f)). Taken together, these data demonstrate that ELK1 promotes LUAD tumor growth *in vivo*.

### 3.5. ELK1 Regulates the EMT in LUAD Cells

**Figure 6:**
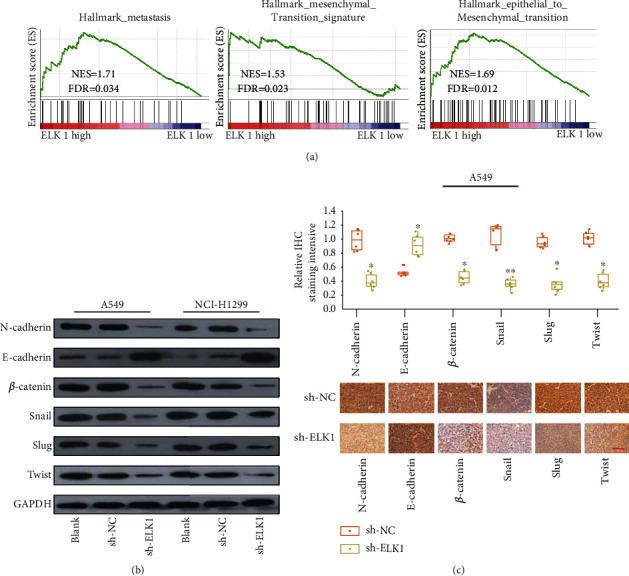
ELK1 is closely related to EMT in the LUAD process. (a) GSEA analysis shows that ELK1 is closely related to tumor metastasis-related genes. (b) Western blotting analysis of EMT-related protein expression changes in A549 and H1299 cell lines after ELK1 knockdown. (c) IHC staining to evaluate the expression of EMT-related protein in tumor tissues of nude mice (scale bar, 200 *μ*m). ^∗^*p* < 0.001 and ^∗∗^*p* < 0.0001.

Our previous studies confirmed that B7-H3 can promote EMT of LUAD cells [16]. Based on the function of B7-H3 as a transcriptional regulator, we next investigated whether ELK1 also affects EMT in LUAD cells. To understand the association of ELK1 with EMT, we conducted GSEA on the TCGA-LUAD dataset. We found that ELK1 was closely related to metastasis, a mesenchymal transition signature, and EMT (Figure 6(a)).

EMT is known to be the key process that occurs before tumor cell metastasis [17, 18]. Therefore, we further analyzed the relationship between ELK1 and EMT in A549 and H1299 LUAD cells. Relative to control, ELK1 knockdown significantly upregulated E-cadherin (epithelial marker), but downregulated N-cadherin (mesenchymal marker); moreover, the levels of EMT-associated transcription factors (Snail, Twist, Slug) dramatically decreased upon ELK1 knockdown (Figure 6(b)).

In addition, we detected the expression of EMT-associated molecules within the xenografts through IHC. According to Figures 6(c), E-cadherin expression increased after ELK1 was knocked down, whereas the expression of N-cadherin, *β*-catenin, Snail, Slug, and Twist levels was decreased. Therefore, these results clearly indicate that ELK1 promotes tumorigenic development in LUAD cells.

### 3.6. ELK1 Promotes the Malignant Biological Behavior of LUAD Cells through B7-H3

**Figure 7:**
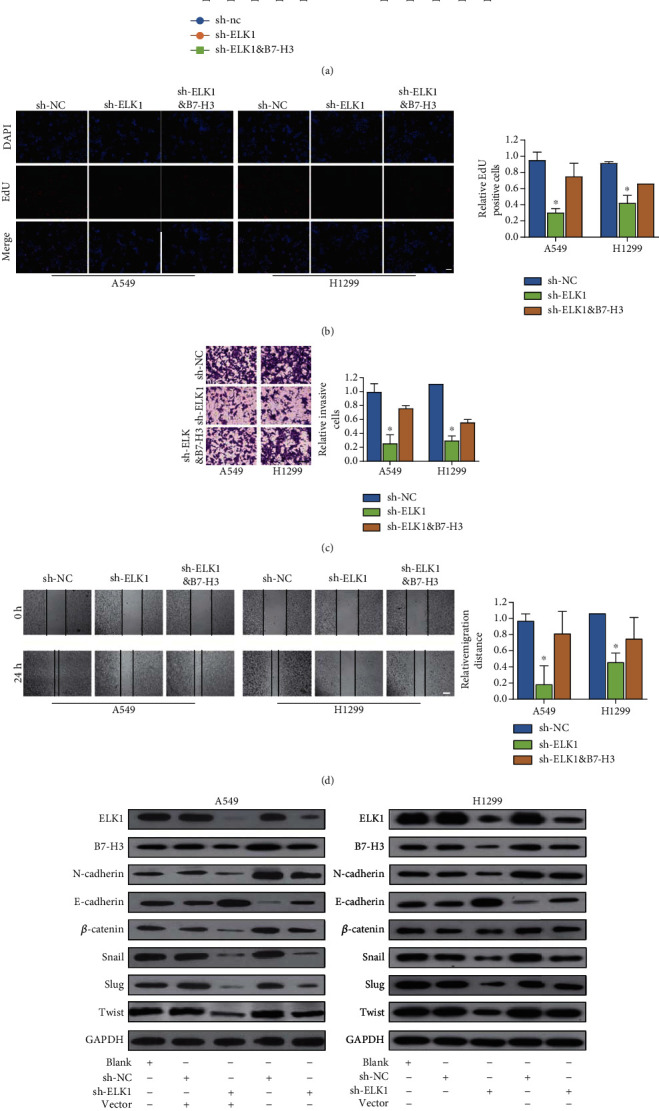
ELK1 plays a key role in the form of relying on B7-H3 in the LUAD process. Overexpression of B7-H3 in ELK1 knockdown A549 and H1299 cells, CCK8, EdU, Transwell, wound healing, and western blotting evaluated the proliferation, invasion, migration, and changes of EMT-related protein, respectively. (a–e) Overexpression of B7-H3 rescued the reduction of LUAD cell proliferation, invasion, migration, and EMT caused by ELK1 knockdown. ^∗^*p* < 0.001.

To further verify that ELK1 participates in LUAD development by affecting the transcription of B7-H3, we conducted rescue experiments using A549 and H1299 cell lines. We transfected sh-NC, sh-ELK1, and sh-ELK1, as well as B7-H3, into these cells and then conducted CCK-8, EdU, Transwell, and wound healing analyses, along with the detection of EMT-related molecules. As shown in Figures 7(a) and 7(b), ELK1 knockdown resulted in reduced proliferation ability, while overexpression of B7-H3 effectively abrogated the inhibition of proliferation induced by the ELK1 knockdown. Consequently, overexpression of B7-H3 partially reversed the effect of sh-ELK1 on cell migration and invasion (Figures 7(c) and 7(d)). Western blot revealed that the increase of E-cadherin and the decrease of N-cadherin, *β*-catenin, Snail, Slug, and Twist induced by ELK1 knockdown were abrogated upon the overexpression of B7-H3 (Figure 7(e)). In summary, ELK1 enhances LUAD cell growth, EMT, migration, and invasion, which is dependent on B7-H3.

## 4. Discussion

B7-H3 has been proven to be a potential therapeutic target for a variety of malignant solid tumors [19]. Our previous research results showed that B7-H3 plays a role in promoting LUAD carcinogenesis and is closely related to tumor cell EMT [16]. However, the regulatory mechanism upstream of B7-H3 remained unclear. In other words, we did not know why B7-H3 is upregulated in LUAD. Transcriptional regulation is a ubiquitous biological phenomenon in mammals. Transcription factors relate to some pathophysiological processes, such as tumorigenesis. The present work shows that ELK1 was a candidate transcriptional regulator for B7-H3.

ELK1 belongs to ETS oncogene family in multiple types of malignancies and plays a role in transcriptional regulation through its ETS DNA-binding domain [20]. Moreover, ELK1 was reported to be a part of a ternary complex factor, and it can be phosphorylated by the MAPK cascade [21]. It is well known that ELK1 has a certain function in different biological processes, like the regulation of cell proliferation, differentiation, apoptosis, and tumorigenesis [22]. In glioblastoma, phosphorylated ELK1 is abundant at the *GDH1* promoter and activates its transcription, promoting glutamine metabolism [23]. As a transcription factor, ELK1 can trigger downstream target oncogenes, including c-Fos, and promote the growth of bladder cancer cells with functional androgen receptors [24]. To the best of our knowledge, research on ELK1 in lung cancer is rare. According to our results, ELK1 showed high expression in LUAD tissues and it is related to the poor prognosis of the patients. In addition, we found that the expression level of ELK1 in LUAD cell lines is generally upregulated. The above findings suggested ELK1 may be involved in the development of LUAD. ELK1 also enhanced activity of the B7-H3 promoter, thereby regulating LUAD cell malignant behaviors. We confirmed this phenomenon through *in vivo* and *in vitro* studies. Therefore, our findings clarify the hitherto unexplored mechanism of ELK1 and B7-H3 in the progression of LUAD.

The invasion and metastasis of tumor cells comprise a series of complex processes, and EMT is an extremely important event in this process. Epithelial cells transform into mesenchymal cells, which is followed by rearrangement of the cytoskeleton, changes in cell polarity, and the formation of new cell-matrix adhesions [25–27]. We have confirmed that the downstream target gene B7-H3 of the transcription factor ELK1 can promote EMT in LUAD cells [16]. Previous studies have shown that B7-H3 overexpression can promote the occurrence of EMT in gliomas through the JAK2/STAT3/Slug signaling pathways [28]. A single-cell sequencing of head and neck squamous cell carcinoma revealed that B7-H3 reshaped the immune microenvironment to promote EMT [29]. In this study, we discovered that ELK1 overexpression promoted EMT, while ELK1 knockdown led to decrease of EMT. In addition, overexpression of B7-H3 could partially reverse the inhibitory effect of ELK1 knockdown on EMT. Therefore, ELK1 regulated EMT partly through B7-H3. It is worth to mention that ELK1 may also affect EMT through other mechanisms. As an important transcription factor, ELK1 may modulate genes which could be involved in EMT. This may explain why the rescue experiments only partially reversed EMT. There are few reports on the association between ELK1 and EMT. In osteosarcoma, Cyr61 promotes EMT and the metastasis of tumor cells through the Raf-1/MEK/ERK/Elk-1/TWIST-1 signaling pathway [30]. During the formation of pulmonary fibrosis, ELK1 transcriptionally regulates the expression of ZC3H4 to promote the silica-induced EMT program [31]. Further, the EMT process is related to many cellular and molecular events, including the downregulation of adhesive epithelial markers and the upregulation of mesenchymal markers. Therefore, we tested the relationship between ELK1 and EMT-related molecules in LUAD through *in vivo* and *in vitro* experiments. We confirmed that ELK1 enhanced EMT in LUAD cells through modulating B7-H3 expression. There are some limitations of the study. The exact mechanism of how B7-H3 induces EMT in LUAD still needs further research and the other mechanisms that ELK1 regulating EMT should be explored in depth.

## 5. Conclusions

**Figure 8:**
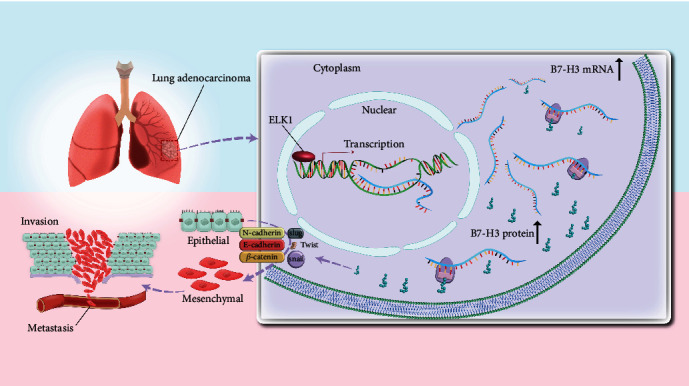
Schematic diagram of the mechanism.

To sum up, the current work illustrates the mechanism by which B7-H3 upregulation regulates LUAD. In short, the transcription factor ELK1 exerts a cancer-promoting effect by binding to the B7-H3 promoter region and stimulating its transcriptional activity, especially promoting EMT (Figure 8). In addition, ELK1 shows high expression within LUAD, which predicts a dismal prognostic outcome for LUAD patients; accordingly, ELK1 could be used as a candidate prognostic biomarker and therapeutic target for LUAD.

## Figures and Tables

**Table 1:** ELK1 expression and clinicopathological characteristics of patients with LUAD.

Features	*n*	ELK1 expression		*χ* ^2^	*P* value
		Low	High		
	90	41	49		
Age (years)				0.045	0.832
≥60	45	20 (48.8)	25 (51.0)		
<60	45	21 (51.2)	24 (49.0)		
Gender				1.994	0.158
Male	49	19 (46.3)	30 (61.2)		
Female	41	22 (53.7)	19 (38.8)		
Smoking				0.231	0.631
Yes	46	23 (56.1)	25 (51.0)		
No	44	18 (43.9)	24 (49.0)		
Differentiation				0.637	0.425
Well/moderate	37	15 (36.6)	22 (44.9)		
Poor	53	26 (63.4)	27 (55.1)		
Tumor size				0.110	0.740
≤3cm	40	19 (46.3)	21 (42.9)		
>3cm	50	22 (53.7)	28 (57.1)		
Lymph node metastasis				9.545	0.002
Yes	51	16 (39.0)	35 (71.4)		
No	39	25 (61.0)	14 (28.6)		
TNM stage				5.443	0.020
I-II	54	30 (73.2)	24 (49.0)		
III-IV	36	11 (26.8)	25 (51.0)		

## Data Availability

Additional data in the present work are available in the manuscript and supplementary materials, upon reasonable request of the corresponding authors.

## Supplementary Materials

Supplementary Materials Table S1: target sequence of ELK1 sh-RNA. Table S2: primers for RT-qPCR.
