# Supplementary material for: ELK1 Promotes Epithelial-Mesenchymal Transition and the Progression of Lung Adenocarcinoma by Upregulating B7-H3
Source: Oxid Med Cell Longev. 2021 Dec 21;2021:2805576. doi: 10.1155/2021/2805576 (PMC8714344; doi:10.1155/2021/2805576)
Supplement: Supplementary Materials — Table S1: target sequence of ELK1 sh-RNA. Table S2: primers for RT-qPCR. [file 2805576.f1.docx]

**Table S1. Target sequence of ELK1 sh-RNA**

| **sh-ELK1** | **Target sequence（5’-3’）** |
| --- | --- |
| shRNA1 | GGGCCTTGCGGTACTACTATGACAA |
| shRNA2 | CGGTACTACTATGACAAGAACATCA |
| shRNA3 | GACAAGAACATCATCCGCAAGGTGA |

**Table S2 Primers for RT-qPCR**

| **Gene** | **Sequence (5’-3’)** |
| --- | --- |
| ELK1 | F: CAGCCAGAGGTGTCTGTTACC |
|  | R: GAGCGCATGTACTCGTTCC |
| B7-H3 | F: GGGTGCGAATGGCACCTACAGC |
|  | R: TGATCTTTCTCCAGCACACG |
